# High Nasal Carriage of MRSA-*mec*C in Wild Rabbits in the Iberian Peninsula: a Wildlife Reservoir?

**DOI:** 10.1007/s00248-026-02713-6

**Published:** 2026-02-10

**Authors:** Carmen González-Azcona, Fernanda Solano-González, Saúl Jiménez-Ruiz, Nuno Santos, Irene Marañón-Clemente, Tamara Álvarez-Gómez, Paula Eguizábal, Carla Andrea Alonso, Daniel Benito, Myriam Zarazaga, Carmen Torres, Carmen Lozano

**Affiliations:** 1 Area of Biochemistry and Molecular Biology, One-Health-UR Research Group, University of La Rioja, Logroño, Spain; 2 Departamento de Sanidad Animal, Grupo de Investigación en Sanidad Animal y Zoonosis (GISAZ), UIC Zoonosis y Enfermedades Emergentes ENZOEM, Universidad de Córdoba, Córdoba, Spain; 3 Grupo Sanidad y Biotecnología (SaBio), Instituto de Investigación en Recursos Cinegéticos IREC (UCLM-CSIC-JCCM), Universidad de Castilla-la Mancha, Ciudad Real, Spain; 4 Centro Investigação Biodiversidade e Recursos Genéticos (CIBIO), Laboratório Associado (InBIO), Universidade do Porto, Vairão, Portugal; 5 BIOPOLIS Program in Genomics, Biodiversity and Land Planning, CIBIO, Vairão, Portugal; 6 Servicio de Análisis Clínicos, Laboratorio de Microbiología, Hospital San Pedro, Logroño, Spain

**Keywords:** *S. aureus*, Wild-rabbit, MRSA, Reservoir, CC130, CC398

## Abstract

*Staphylococcus aureus* is a commonly found bacteria on the skin and mucous membranes of humans and animals. It can act as an opportunistic pathogen causing multiple infections of diverse severity. Moreover, *S. aureus* can acquire resistance to multiple antibiotics, with methicillin-resistant *S. aureus* (MRSA) being a significant global health concern. European wild rabbit (*Oryctolagus cuniculus*) is an important species in Iberian ecosystems and can serve as reservoirs for zoonotic bacteria. In this study, 472 nasal samples from wild rabbits in Spain and Portugal were analyzed to determine the prevalence, antimicrobial resistance, and molecular characteristics of *S. aureus*. Overall, *S. aureus* was detected in 13.6% of animals, with higher prevalence in animals of Spain (27.4%) than in those of Portugal (6.2%). MRSA was found in 51 samples (10.8%), predominantly carrying the *mec*C-*agr*III-SCC*mec*XI genes associated with CC130, and three isolates carried *mec*A-*agr*I-SCC*mec*V and belonged to CC398. Resistance to penicillin (92.1%), cefoxitin (79.6%), ciprofloxacin (7.9%), tetracycline (5.7%), erythromycin (2.3%) and gentamicin (1.1%) was observed. A marked intra-host diversity was detected and different *S. aureus* isolates were observed in the same sample in 35.4% of the positive rabbits. Virulence genes *luk*ED and *etd*2 were frequent, whereas the *scn* human-adaptation marker (IEC system) was present in 33.0% of the eighty-eight non repetitive *S. aureus* isolates. These results suggest that Iberian wild rabbits may constitute a relevant reservoir of MRSA-*mec*C-CC130, highlighting their potential role in One-Health dynamics, warranting continued wildlife surveillance.

## Introduction

*Staphylococcus aureus* (*S. aureus*) is present as a commensal on the skin, the nose and mucous membranes of healthy humans. However, it is also an opportunistic pathogen that can cause multiple infectious diseases of diverse severity [1]. Moreover, *S. aureus* colonization is common in different types of animals including livestock (pigs, cows or poultry) [2], pets (dogs, cats, horses) [3], and wild mammals or birds (wild ruminants, small mammals, cervids, pelicans, among others) [2–8]. In animals, this microorganism can cause bovine mastitis, poultry bacterial infection, rabbit suppurative dermatitis, and other diseases [2, 9]. The pathogenicity of *S. aureus* is due to the presence of various virulence factors, as the toxic-shock syndrome toxin (TSST) or the Panton-Valentine leukocidin (PVL), among others. Moreover, *S. aureus* also has a great capacity to acquire multiple resistance mechanisms to several antimicrobial agents, which limits the therapeutic options [10]. Antibiotic-resistant *S. aureus -*particularly methicillin-resistant isolates- represents a global health problem.

Methicillin-resistant *S. aureus* (MRSA) is an important cause of morbidity and mortality throughout the world [11]. Methicillin-resistance is commonly associated with *mec*A gene in both humans and animals [12]. In this regard, livestock-associated (LA) methicillin-resistant *S. aureus* (LA-MRSA) predominantly carries the *mec*A gene and belongs to clonal complex (CC) 398. MRSA CC398 gained much attention in the last years because, apart from colonizing farm animals, it became a frequent pathogen in humans, mainly -but not always- in contact with livestock [2, 11, 13].

An additional *mec* gene, known as *mec*C, was discovered during an epidemiological investigation of bovine mastitis [14]. Similar to *mec*A, the *mec*C gene was found to be present inside staphylococcal cassette chromosome *mec* (SCC*mec*) [15]. The *mec*C gene has been identified in several MRSA lineages, mainly related to animals, such as CC130, CC49, ST425, CC599, and CC1943 [16]. CC130 was initially associated with methicillin-susceptible *S. aureus* (MSSA) isolates from animals, but in recent years, it has gained interest because of its relationship with the methicillin-resistance determinant *mec*C and the SCC*mec* type XI [2, 5–7, 10, 12, 16, 17].

Wildlife has been recognized to be an important component for the One-Health approach, as it can be a source of microorganisms that could cause emerging and re-emerging infectious diseases that could spill over to urban settlements and infect humans and domestic animals [18]. Wildlife is not directly exposed to clinical antimicrobial agents but can acquire antimicrobial-resistant bacteria through contact with humans, animals and the contaminated environment [19]. Notably, recent evidence indicates that wildlife does not merely acquire MRSA from humans or livestock; in hedgehogs, zoonotic MRSA-*mec*C lineages appear to predate the antibiotic era, underscoring long-standing ecological reservoirs within One-Health networks [20].

The Iberian Peninsula is a territory of diverse wildlife and natural resources. Several studies in Spain and Portugal have determined the prevalence and characteristics of *S. aureus* obtained from different wild mammals [21–24]. The European wild rabbit (*Oryctolagus cuniculus*) is a very important species in the Iberian Peninsula because it plays a very relevant role in the ecology of the regions in which it inhabits and can enter into the food chain through hunting activities. In a previous study, the nasal microbiota of 147 European wild rabbits was analysed, and *S. aureus* isolates were obtained in 13.6% of the samples (20 samples), being most of the recovered *S. aureus* isolates resistant to methicillin (22 of 28 *S. aureus* isolates, 78.6%) [18]. To deepen in the characterization of *S. aureus* in this host, we enlarged the number of wild rabbits tested to determine the prevalence of *S. aureus* and MRSA in nasal samples of European wild rabbits in the Iberian Peninsula, as well as to characterize the isolates obtained and assess intra-host diversity.

## Methods

### Sample collection, Transport and Preservation

A total of 529 nasal samples of European wild rabbits were obtained in different areas of the Iberian Peninsula from November 2022 to January 2025, and 472 were included in this study (57 samples were excluded due to heavy contamination with *Bacillus*). Sampling was as follows: (a) 164 samples of wild rabbits from eight provinces of Spain that were classified in three zones: North (La Rioja, Zaragoza, Navarra, Álava and Burgos), Centre (Toledo and Cuenca) and South (Jaén and Granada); and (b) 308 samples of wild rabbits from two zones of Portugal: Centre (Santarém) and South (Beja). Samples were obtained from: (a) hunting activities (*n* = 164, all from Spain); (b) a longitudinal capture–mark–recapture study in which live rabbits were captured and released after sample collection (*n* = 308, all from Portugal). The study was approved by the ethical committee of the CIBIO’s Animal Welfare and Ethics Review Board (ORBEA/2023_01).

For nasal sampling, sterile cotton-tipped swabs were used and inserted into the nostrils. They were transferred to commercial Amies transport medium tubes and stored at 4 °C or frozen if they were not immediately analysed.

Among the 472 samples used in the present study, it was included: (a) 325 nasal samples that were processed for *S. aureus* recovery; (b) 147 samples analysed in the previous study [18], in which 28 *S. aureus* were recovered, and the isolates were characterized in the present study.

### Bacterial Isolation and Identification

Nasal swab samples were first placed in Brain Heart Infusion broth (BHI, Condalab, Madrid, Spain). An aliquot was subsequently inoculated in BHI broth with 6.5% sodium chloride (NaCl) and then, they were incubated for 24 h at 37 °C. After that, 40 µL of the broth samples were inoculated into three culture media: mannitol salt agar (MSA, Condalab, Madrid, Spain), MRSA chromogenic media (Brilliance^TM^MRSA2/Brilliance™ Staph24, Thermo Fisher), and blood agar (BioMerieux, Madrid, Spain). Plates were incubated for 24 to 48 h at 37 °C for *S. aureus* and MRSA recovery. After overnight growth, up to 8 different colonies per sample (based on morphology, colour and haemolysis) were randomly selected. Colonies were identified by matrix-assisted laser desorption/ionization time-of-flight mass spectrometry (MALDI-TOF; Bruker Daltonics, Bremen, Germany) using the standard extraction protocol recommended by the manufacturer. The protein profile of *E. coli* DH5α peptide was used for calibration purposes. Isolates identified as *S. aureus* were maintained and characterized in this study.

### Antimicrobial Resistance Phenotype and Genotype

The antimicrobial susceptibility phenotypes were determined in *S. aureus* by disk-diffusion method for the following antimicrobial agents (charge of disk in µg): penicillin (1), cefoxitin (30), erythromycin (15), clindamycin (2), gentamicin (10), tobramycin (10), tetracycline (30), ciprofloxacin (5), chloramphenicol (30), linezolid (10), trimethoprim–sulfamethoxazole (1.25 + 23.7) and mupirocin (200). The staphylococcal disk diffusion results were interpreted according to the European Committee of Antimicrobial Susceptibility Testing [25].

Moreover, the following resistance genes were analyzed by PCR and sequencing of obtained amplicons: beta-lactams (*mec*A, *mec*C, *bla*Z and *bla*Z-SCC*mec*XI), erythromycin [*msr*(A) and *mph*(C)], erythromycin-clindamycin [*erm*(A), *erm*(C)], clindamycin [*lnu*(A), *lnu*(B), *lsa*(B) and *vga*(A)], tetracycline [*tet*(K), *tet*(L) and *tet*(M)], gentamicin-tobramycin [*aac*(6´)-le-*aph*(2´´)-la], tobramycin [*ant*(4´)-la], linezolid [*cfr*, *optr*A, *poxt*A], chloramphenicol (*fex*A, *fex*B, *catp*_C194_, *catp*_C221,_*catp*_C223_) and mupirocin (*mup*A) [26].

### Molecular Typing

All *S. aureus* isolates were characterized by *spa*-typing and the obtained sequences were analyzed using Ridom Staph Type software (Ridom GmbH, Münster, Germany). The unique repeat combination was submitted to the Ridom *spa* Server. Thirteen representative isolates, one per *spa*-type detected, were typed by Multi-locus-sequence-typing (MLST). For this aim, PCR/sequencing of seven housekeeping genes was performed to determine the sequence type (ST) and the clonal complex (CC). The remaining isolates were assigned to CC according to their *spa*-types. Additionally, the *agr* and SCC*mec*-typing were performed in all *S. aureus* and MRSA isolates, respectively [27].

### Virulence and Host Adaptation Markers

The presence of the genes encoding virulence factors as Panton-Valentine leukocidin (*lukF/S*-PV), toxic shock syndrome toxin (*tst*), exfoliative-toxins A, B and D (*eta*,* etb* and *etd*2, respectively), and bicomponent pore-forming toxin leukocidin ED (*lukED*) was tested in all *S. aureus* isolates by PCR. Also, the human Immune Evasion Cluster (IEC) genes (*scn*, *chp*, *sak*, *sea*, and *sep*) were investigated by PCR, using the *scn* gene as a marker of the IEC system. Depending on the IEC genes detected, seven IEC types were identified (A–G). The IEC system has been considered as a human adaptation marker [28].

### Statistical Analysis

To evaluate the possible absence or presence of *S. aureus* and MRSA-*mec*C in different areas of the Iberian Peninsula, the Chi-square (to ensure the validity of the results, Fisher’s exact test was used in cases where the sample size was small) and *p*-value were calculated. All calculations were performed in R 4.4.1 (R Core Team, 2025). Statistical significance was set at *p* < 0.05 for analysis.

## RESULTS

### Prevalence of S. *aureus* and MRSA in Nasal Cavities of Wild Rabbits

**Fig. 1:**
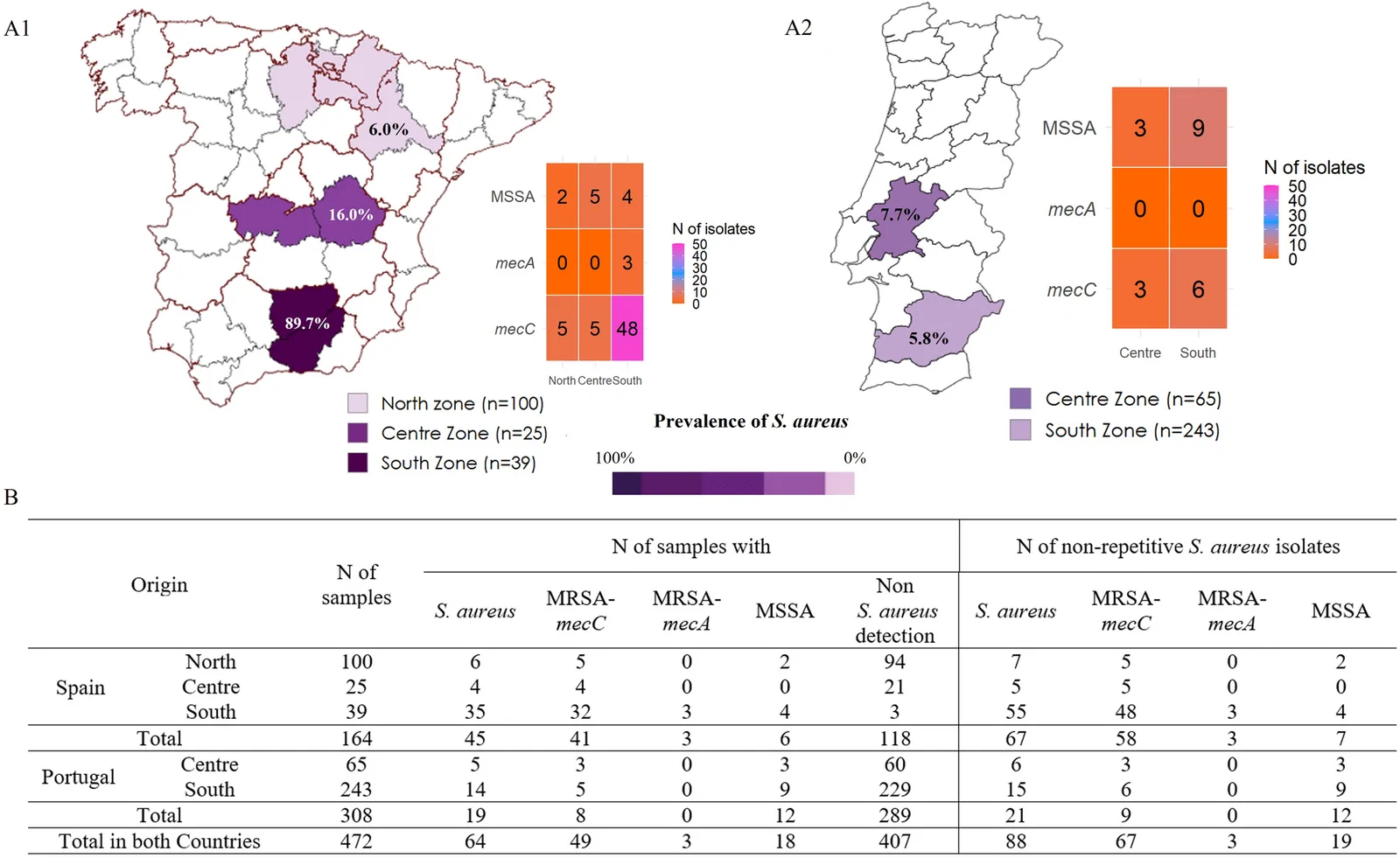
Prevalence and distribution of MRSA-*mec*C, MRSA-*mec*A and MSSA in wild rabbits from the Iberian Peninsula. **A1**) Spain; **A2**) Portugal; **B**) Combined prevalence by region. Colour scale indicates percentage of *S. aureus* positive samples

A total of 64 of the 472 nasal wild rabbit samples carried *S. aureus* isolates (13.6%) (Fig. 1). A higher prevalence of *S. aureus* was detected in samples from Spain (45/164 samples, 27.4%) compared to those of Portugal (19/308 samples, 6.2%). The prevalence of *S. aureus* detected in the different zones is shown in Fig. 1. Significant differences (*p* < 0.05) were detected in areas with the highest rates (southern Spain) and those with the lowest rates (southern Portugal and northern Spain).

Ninety-eight *S. aureus* isolates were initially recovered and identified from the 64 positive samples, detecting between one to three *S. aureus* isolates per sample. After antimicrobial resistance phenotype and *spa*-type determination, non-repetitive *S. aureus* isolates were selected, considering as non-repetitive isolates: one *S. aureus* isolate per sample or more than one if they presented different antimicrobial resistance phenotypes and/or different *spa*-types. So, 88 *S. aureus* isolates were considered as non-repetitive *S. aureus* isolates and were further characterized in this study (Table 1).

Seventy of the 88 *S. aureus* presented a methicillin-resistance phenotype (79.6%, MRSA). MRSA isolates were detected in 51 of the 472 nasal samples (10.8%) and a higher prevalence of MRSA was detected in samples from Spain (43/164 samples, 26.2%) compared to those of Portugal (8/308 samples, 2.6%). A total of 70 non-repetitive MRSA isolates were recovered from the 51 MRSA-positive samples, nine isolates from samples of Portugal and the remaining 61 from samples of Spain (Fig. 1; Table 1).

**Table 1:** Molecular characterization of the 88 non-repetitive *S. aureus* isolates detected

Type of S. aureus	*spa*-type	MLST-CC*	*agr*/SCC*mec*	*N* of samples*	*N* of non-repetitive isolates*	Resistance phenotype (*N* of isolates)*	Resistance genes (*N* of isolates)*	Virulence (*N* of isolates)*	IEC (*N* of isolates)*
MRSA-*mecC*	t843	ST1945-CC130	III/XI	21	22	PEN-FOX ^21^ PEN-FOX-CIP^1^	*bla*Z^22^ *mec*C^22^	*lukED*^18^ *etd*2 ^21^	E^10^, No IEC^12^
	t1535	ST1945-CC130	III/XI	18	21	PEN-FOX^17^ PEN-FOX-ERY^2^ PEN-FOX-CIP^2^	*bla*Z ^17^ *mec*C ^17^ *bla*Z ^2^ *mec*C ^2^ *msr(*A)^2^ *blaZ *^2^ *mec*C ^2^	*lukED*^19^ *etd*2 ^20^	E^3^, No IEC^18^
	t1736	ST1583-CC130	III/XI	12	14	PEN-FOX^11^ PEN-FOX-CIP^1^ PEN-FOX-GEN^1^ PEN-FOX-TET^1^	*bla*Z ^11^ *mec*C ^11^ *bla*Z ^1^ *mec*C ^1^ *bla*Z ^1^ *mec*C ^1^ *aac*(6´)-le-*aph*(2´´)-la^1^ *bla*Z ^1^ *mec*C ^1^ *tet*(L)^1^	*lukED*^13^ *etd*2 ^13^	E^8^, No IEC^6^
	t1773	ST1945-CC130	III/XI	6	6	PEN-FOX^6^	*bla*Z ^6^ *mec*C ^6^	*lukED*^5^ *etd*2^6^	No IEC^6^
	t13275	ST12944-CC130	III/XI	2	2	PEN-FOX^2^	*bla*Z ^2^ *mec*C ^2^	*lukED*^2^ *etd*2^1^	No IEC^2^
	t15608	ST1945-CC130	III/XI	2	2	PEN-FOX^2^	*blaZ* ^2^ *mec*C ^2^	*lukED*^1^ *etd*2^2^	No IEC^2^
MRSA-*mecA*	t1606	ST398-CC398	I/V	1	1	PEN-FOX-TET-CIP^1^	*bla*Z ^1^ *mec*A^1^ *tet*(M)^1^	^−^	No IEC^1^
	t011	ST398-CC398	I/V	2	2	PEN-FOX-TET^2^	*bla*Z ^2^ *mecA* ^2^ *tet*(M)^2^	^−^	No IEC^2^
MSSA	t084	ST15-CC15	II	11	11	PEN^8^ PEN-TET^1^ Susceptible^2^	*bla*Z ^6^ *tet*(K)^1^ ^−^	*lukED*^6^ *etd*2^2^	C^5^, B^2^, No IEC^4^
	t937	ST291-ND	II	2	2	PEN-CIP^2^	*bla*Z ^2^	*lukED*^2^ *etd*2^2^	B^1^, No IEC^1^
	t645	ST1945-CC130	IV	2	2	Susceptible^2^	-	*lukED*^2^ *etd*2^1^	No IEC^2^
	t3750	ST2328-CC133	III	1	1	Susceptible^1^	-	-	No IEC^1^
	t18272	ST8896-ND	IV	1	1	Susceptible^1^	-	*lukED* ^1^	No IEC^1^
	t1736	ST1583-CC130	IV	1	1	Susceptible^1^	-	*lukED*^1^ *etd*2^1^	No IEC^1^

*Abbreviations: CC: clonal complex; CIP, ciprofloxacin; ERY, erythromycin; FOX, cefoxitin; GEN, gentamicin; IEC: Immune-Evasion-Cluster; N: number; ND: not determine; PEN, penicillin; TET, tetracycline.

### Resistance Phenotype and Genotype of *S*. aureus Isolates

**Fig. 2:**
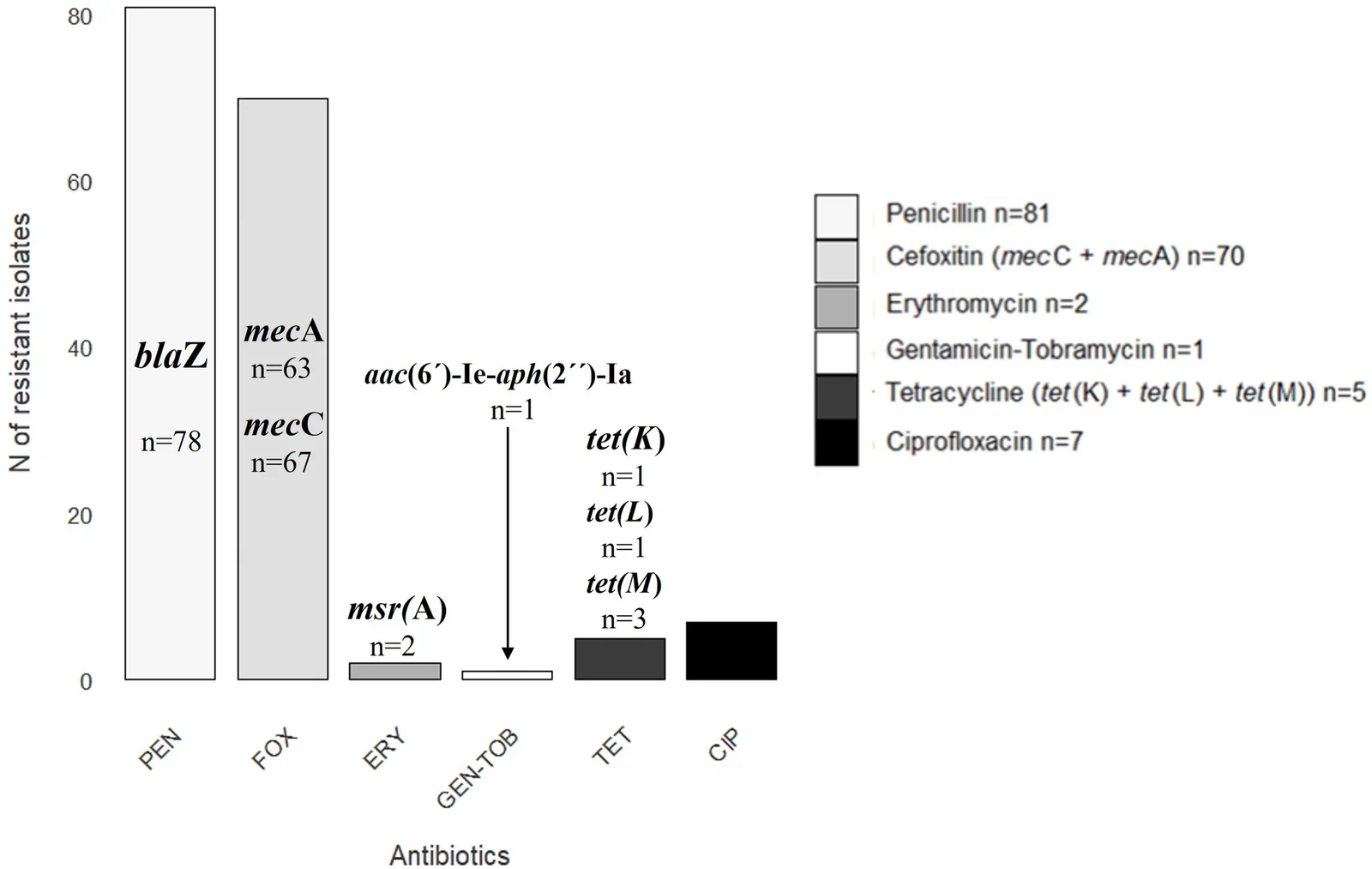
Antimicrobial resistance phenotypes and genotypes detected among the 88 non-repetitive *S. aureus* isolates from wild rabbits. PEN: penicillin; FOX: cefoxitin; ERY: erythromycin; GEN: gentamicin; TOB: tobramycin; TET: tetracycline; CIP: ciprofloxacin. Numbers of isolates are indicated

Among the 88 non-repetitive *S. aureus* isolates, seven (7.9%) were susceptible to all the antimicrobial agents tested. The remaining isolates showed resistance to (n. of isolates/%): penicillin (81/92.1%), cefoxitin (70/79.6%), ciprofloxacin (7/7.9%), tetracycline (5/5.7%), erythromycin (2/2.3%), and gentamicin (1/1.1%). Resistance to linezolid, chloramphenicol and mupirocin was not found among the *S. aureus* isolates (Table 1; Fig. 2).

Among the 70 MRSA isolates recovered, 67 harboured the gene *mec*Cand three the gene *mec*A. The 67 MRSA-*mec*C isolates were obtained from 49 of the 472 samples (10.4%), representing variable percentage among the samples of the different zones (2.1%−82.1%): 2.1% and 4.6% in South and Centre Portugal and 5.0%, 16.0% and 82.1% in North, Centre and South Spain, respectively (Fig. 1). Significant differences (*p* < 0.05) were also identified in relation to MRSA-*mec*C detection in the region with the highest rate (southern Spain) and the lowest rate (southern Portugal). The three MRSA-*mec*A isolates were obtained from three samples collected in southern Spain. Eleven MRSA isolates exhibited resistance to ciprofloxacin, tetracycline, erythromycin or gentamicin apart from penicillin and cefoxitin. One isolate showed a multi-drug-resistant (MDR) phenotype (resistance to penicillin, cefoxitin, tetracycline and ciprofloxacin), and it was of the CC398 lineage.

The resistance genes found were (number of isolates): *msr(*A) (*n* = 2), *tet(*M) (*n* = 3), *tet(*L) (*n* = 1) and *aac*(6´)-le-*aph*(2´´)-la (*n* = 1); moreover, five isolates showed resistance to ciprofloxacin (Table 1; Fig. 2).

Among the 18 methicillin-susceptible *S. aureus* (MSSA) isolates, seven were susceptible to all antibiotics tested. The remaining eleven presented resistance to penicillin/*bla*Z (*n* = 11/8), tetracycline/*tet(*K) (*n* = 1) and ciprofloxacin (*n* = 2) (Table 1; Fig. 2).

### Molecular Characterisation of *S*. aureus Isolates

Thirteen different *spa*-types were detected. Among MRSA-*mec*C isolates the following *spa*-types were identified (n. of isolates): t843 (22), t1535 (21), t1736 (14), t1773 (6) and t13275/t15608 (2). Sixty-seven MRSA-*mec*C isolates belonged to CC130 (ST1945, ST1583 and ST2944) and presented the SCC*mec* type XI and *agr* type III. The MRSA-*mec*A isolates presented the *spa*-types t011 (2 isolates) and t1606 (1 isolate), and the three ones belonged to ST398-CC398 and showed the SCC*mec* type V and *agr* type I (Table 1).

A high diversity of lineages was identified among the 18 MSSA isolates, where three CCs were detected associated with the following *spa*-types (number of isolates): CC15-t084 (11), CC130 [t645 (2) and t1736 (1)], and CC133-t3750 (1). Moreover, two isolates belonged to ST291-t937 that is a double locus variant of ST398 and one isolate to ST8896-t18272. These isolates presented *agr* types II (13 isolates), III (1 isolate) and IV (4 isolates) (Table 1).

### IEC and Virulence Gene Content

**Fig. 3:**
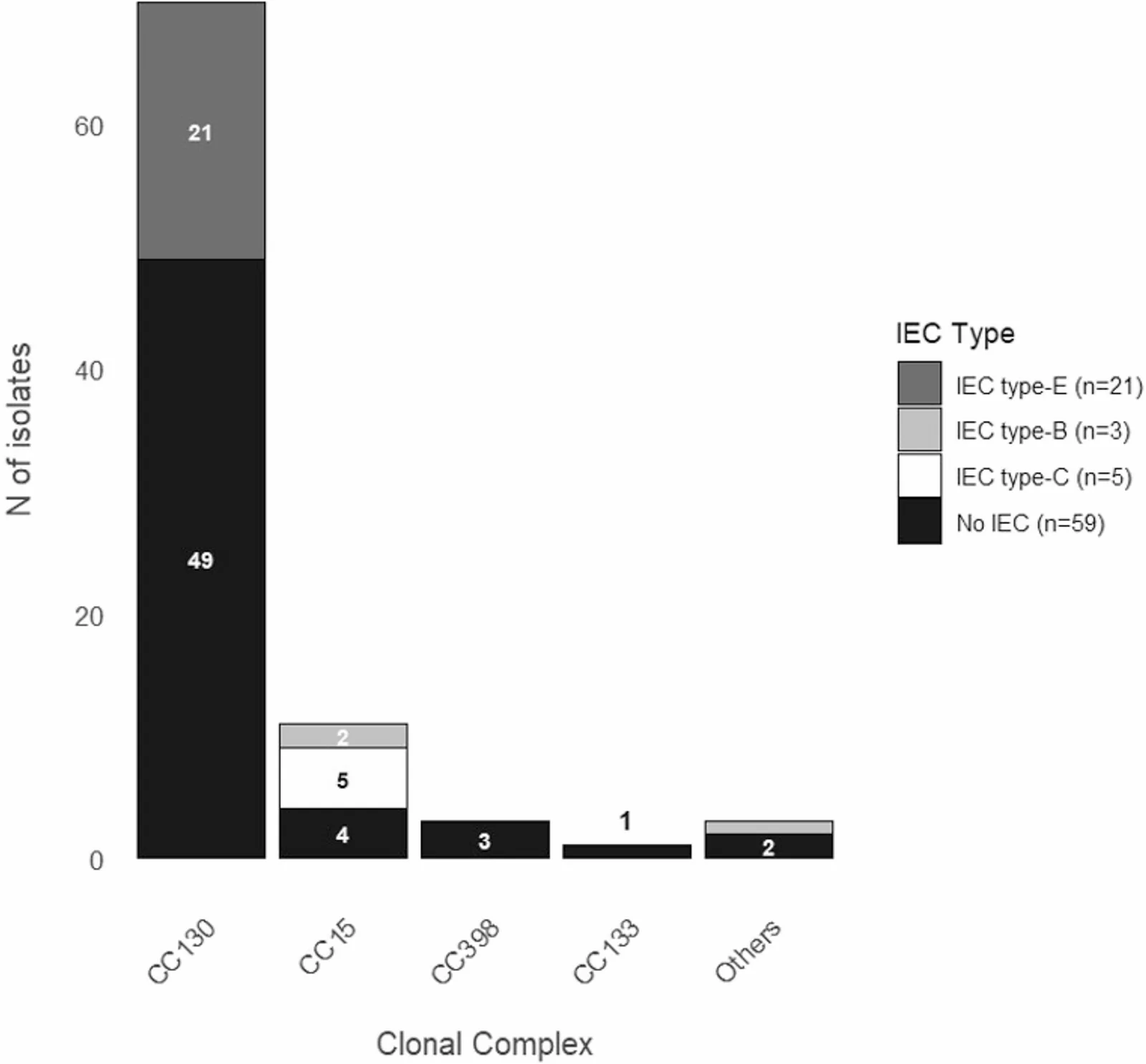
Detection of immune-evasion cluster (IEC) types (A-E) across the different clonal complexes (CC) of *S. aureus*

The gene *scn* (marker to the human IEC system) was identified in 29 of the 88 *S. aureus* isolates corresponding to 21 of the 70 MRSA isolates (30%) and 8 of the 18 MSSA isolates (44.4%) (Table 1; Fig. 3). Fifty-nine isolates were IEC negative (67.1%) and belonged to CC130 (49/70, 70%), CC15 (4/11, 36.4%), CC398 (3/3, 100.0%), ST291 (1/2, 50%), CC133-ST8896 (1/1, 100.0%). All isolates that presented the IEC type E were MRSA-*mec*C isolates (CC130) (21 isolates), and types B and C were MSSA isolates (8 isolates, CC15 and ST291) (Table 1; Fig. 3).

None of the isolates carried the virulence genes *tst*,* lukS*/*F*-PV, *eta* or *etb*. Sixty-nine isolates were positive for *etd2* gene and 70 were positive for *lukED* (Table 1).

### Variety of non-repetitive *S*. aureus Isolates among the Animals Tested

**Fig. 4:**
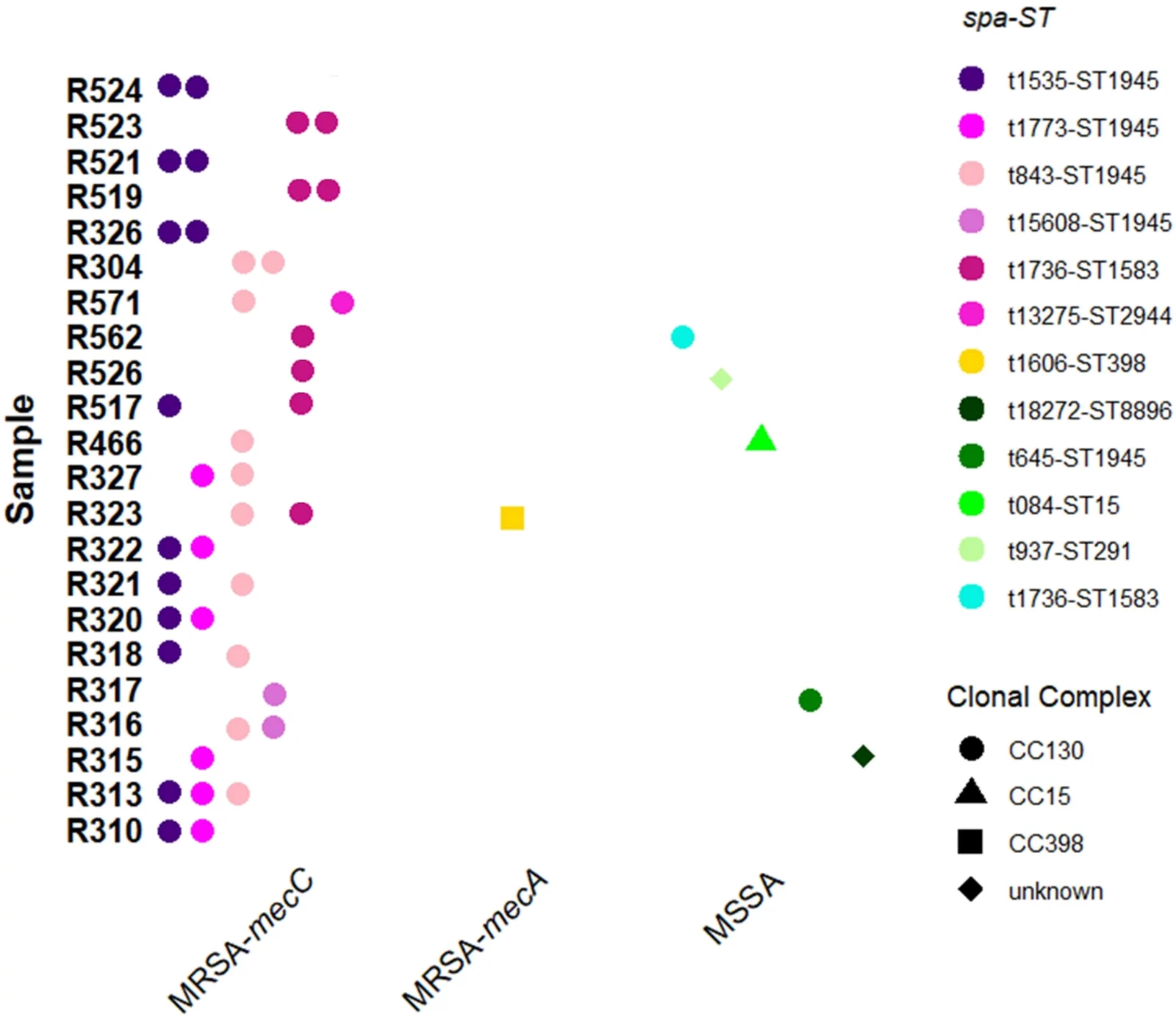
Samples in which more than one non-repetitive *S. aureus* isolate was found

Non-repetitive *S. aureus* isolates were considered as those that presented a different *spa-*type, resistance phenotype or, in the case of MRSA, methicillin-resistance gene (*mec*A or *mec*C). Thus, more than one non-repetitive *S. aureus* isolate was detected in a total of 23 of the 64 positive animals (35.9%): 14 samples carried *S. aureus* isolates with two different *spa*-types (the most frequent associations were t1773-t1535 and t1535-t843), and two samples carried three different *spa*-types (t843-t1606-t1736 and t843-t1535-t1773). Moreover, in one sample, both MRSA-*mec*C and MRSA-*mec*A isolates were identified, and seven samples contained *S. aureus* isolates with the same *spa*-type but different antibiotic resistance phenotypes (Table 1; Fig. 4).

## Discussion

The prevalence of *S. aureus* in European wild rabbit nasal samples of Spain and Portugal was 13.8%, and it is in the range compared to some previous studies in which these animals were analysed in the Iberian Peninsula (8% and 22%, respectively) [10, 23]. However, a higher value (41.3%) was obtained in lagomorphs in a work performed in another Spanish region (Valencia) [29]. Considering other wild animals, *S. aureus* was found in 12.9% in small mammals and 35.8% in ungulates [5, 30]. In other European countries, different *S. aureus* prevalence values have been observed, for example, 45.5% in wild boars (*Sus scrofa*) or 15.3% in rodents/shrews [31, 32]. In this regard, it is noteworthy that *S. aureus* was detected in 92.1% of the hedgehog samples tested in France [33]. Indeed, hedgehogs have been proposed as an important reservoir of MRSA isolates carrying the resistance mechanism *mec*C, as a high prevalence of MRSA-*mec*C isolates (64.0% and 38.0%) was detected in two studies in Europe and New Zealand [20, 34].

In our study, 70 of the total *S. aureus* isolates (79.5%) were methicillin-resistant (MRSA), 67 of them containing the *mec*C gene (76.1%). Similar results were obtained in the study performed in Valencia, in which 63.3% of the *S. aureus* were MRSA and all of them, except one, carried the *mec*C gene [29]. In the case of hedgehogs, it was speculated that the abundance of MRSA-*mec*C was enhanced by antibiotic production by *Trichophyton erinacei.* This fungus produces two beta-lactam antibiotics, providing a selective environment in which MRSA-*mec*C isolates have an advantage over susceptible isolates [20]. *T. erinacei* is also an important dermatophyte that causes infections in small mammals, and it is also known to colonize rabbits [35]. Given the high detection rate of MRSA-*mec*C isolates found in our study and in others [29], it seems that this species can also constitute an important reservoir of this resistance mechanism. It would be interesting to analyze whether this fungus or another one might be related to its emergence and/or persistence in wild rabbits. The *mec*C gene has also been detected in other species (mainly wild), such as storks (*Ciconia ciconia*), red deer (*Cervus elaphus*), or other mammals [4–6, 10]. MRSA-*mec*C has also been detected as an agent causing infections in humans [12]. The possible introduction of MRSA-*mec*C isolates through hunting and their entry through the food chain must be taken into account. The recent genomic evidence of direct transmission of MRSA-*mec*C (ST130-t843) between a horse and a veterinarian in Hungary underpins the zoonotic potential of wildlife -and livestock-associated reservoirs and underscores the necessity of cross-sectoral surveillance frameworks [36]. Moreover, the *mec*C gene has been not only detected in *S. aureus* isolates but also in coagulase-negative-staphylococci (CoNS) [37]. CoNS may also act as reservoirs for this resistance mechanism, and its transfer to more virulent species such as *S. aureus* should be analyzed.

Although MRSA-*mec*C often displays limited non-β-lactam resistance, reports from wildlife and livestock indicate variable MDR patterns; thus, local resistomes should be interpreted cautiously [38]. Multidrug resistance was not detected in any of our MRSA-*mec*C isolates; however, eleven of them were resistant (in addition to beta-lactams) to ciprofloxacin, tetracycline, erythromycin or gentamicin.

All MRSA-*mec*C isolates belonged to CC130 clonal complex (ST1583/ST1945/ST2944), which is related to livestock and wild animals. This clonal lineage is the one most commonly associated with MRSA-*mec*C isolates and it has been frequently detected in wild animals [2, 10, 20]. The *spa*-types most frequently detected in CC130 isolates have been t843, t1535 and t1736 [16, 17],. The mentioned *spa*-types and others were found in our study (n. of isolates): t843 (22), t1535 (21), t1736 (14), t1773 (6), t13275 (2), and t15608 (2). The *spa*-types t843 and t1535 were the most prevalent, consistent with previous findings [39, 40]. Nevertheless, MRSA-*mec*C isolates belonging to other CCs, such as CC49, ST151, ST425, CC599 or CC1943, have been also described in different hosts and in environmental samples [2, 16, 20, 39, 41].

A recent systematic phylogenomic review exploring *mec*C-mediated methicillin-resistance across non-*aureus* staphylococci and *Mammaliicoccus* raises the hypothesis of a wildlife origin and broad host-range dissemination of the *mec*C trait, challenging the traditional livestock-only reservoir model [38].

Only three *S. aureus* isolates carried the *mec*A gene. Although to a lesser extent than *mec*C, MRSA-*mec*A isolates have been also identified in wild animals in prevalence from 0.70% to 7.7% [21, 29, 40, 42]. Our MRSA-*mec*A isolates belonged to CC398. The CC398 clonal complex was originally associated with pigs [43], but is widely distributed, in much lower prevalence, in other animals: wild ruminants, vultures and wild boar [2, 7, 44] and occasionally in humans (especially in pig-associated professionals) [45]. ST398 is the most frequent in CC398 [4] and the typical *spa*-types are t011, t034 and t1451 [21, 26, 46, 47]. In this study, the three isolates belonged to ST398. Two presented the *spa*-type t011 and one t1606. CC398 isolates usually present multidrug resistance [27, 48]. Only one of the CC398 isolates was multidrug resistant, being only resistant in addition to beta-lactams, to tetracycline and ciprofloxacin. Moreover, none of the CC398 isolates presented IEC. Thus, the three MRSA-*mec*A CC398 isolates presented characteristics considered as animal clade CC398 [2, 13, 43, 49].

Related to MSSA isolates, they belonged to different *spa*-types and ST-CC. Most of the MSSA isolates belonged to CC15 (61.1%), a common CC found in nasal samples of different animals and food samples such as poultry meat, dogs, cats or cows [27, 50–52], reporting low resistance burdens in this lineage. The remaining isolates belonged to CC130, CC133, ST291 and ST8896. MSSA-CC130 is commonly found in cattle and other livestock animals and can be an important cause of disease [16]. The *spa*-types of the three MSSA-CC130 were t645 and t1736. The *spa*-type t1736 has been also described in MRSA-*mec*C isolates [33, 34].

In addition to antibiotic resistance, *S. aureus* can carry a wide range of virulence genes capable of increasing the pathogenic potential of the isolates. In this study, the *lukS*/*F*-PV *tst*, *eta*, and *etb* genes were not detected in any isolate. In contrast, most isolates harbored the *luk*ED (79.5%) and *etd*2 (78.4%) genes, with 98.6% of MRSA-*mec*C-CC130 isolates testing positive for both markers. Previous studies have similarly highlighted an association of *luk*ED and *etd*2 with the CC130 lineage [5, 6, 16]. Overall, these findings support that the presence of *luk*ED and *etd*2 constitutes a stable characteristic of the CC130 lineage across both human- and animal-derived isolates, underscoring their potential relevance as virulence markers within this clade.

Finally, important intra-host diversity was detected, and different *S. aureus* isolates were observed in the same sample in 35.9% of the positive rabbits. Remarkably, one of the animals carried simultaneously MRSA-*mec*C and MRSA-*mec*A isolates and seven samples contained *S. aureus* isolates with similar *spa*-type but different antibiotic resistance phenotypes, indicating how selective pressure may be promoting the acquisition of different resistance mechanisms in isolates of the same genetic lineage. Finally, none of the *S. aureus* isolates obtained showed resistance to mupirocin, linezolid or chloramphenicol. The absence of linezolid resistance is particularly relevant, as this antibiotic is a last-line option against MRSA.

This study provides a large-scale, Iberia-wide assessment and reveals substantial intra-host *S. aureus* diversity, including concurrent *mec*C (formerly called *mec*A_LGA251_)-CC130 and *mec*A-CC398 carriage. By identifying European wild rabbits as effective One-Health sentinels for MRSA-*mec*C circulation, our findings suggest that wildlife hosts can sustain stable and independent reservoirs of clinically relevant resistance lineages. The predominance of SCC*mec*XI-CC130, persisting in the absence of direct antimicrobial selective pressure, highlights that methicillin-resistance can be maintained and disseminated through ecological and evolutionary mechanisms beyond antibiotic use.

In this context, the widespread circulation of *mec*C-positive-CC130 in wildlife highlights a cryptic yet epidemiologically relevant transmission cycle at the wildlife-environment-livestock-human interface. Given the ecological connectivity of wild rabbits with food-producing systems and the food chain, these reservoirs represent an underrecognized pathway for the reintroduction of MRSA-*mec*C into human-associated settings. Collectively, our findings underscore the need to integrate wildlife into One-Health antimicrobial resistance surveillance frameworks to fully capture the diversity and transmission dynamics of methicillin-resistance determinants.

## Data Availability

All the data derived from this study were comprehensively presented in this article. However, additional information may be requested from the corresponding author.
